# Analysis of a Smartphone-Based Architecture with Multiple Mobility Sensors for Fall Detection

**DOI:** 10.1371/journal.pone.0168069

**Published:** 2016-12-08

**Authors:** Eduardo Casilari, Jose Antonio Santoyo-Ramón, Jose Manuel Cano-García

**Affiliations:** Departamento de Tecnología Electrónica, Universidad de Málaga, Malaga, Spain; Universita degli Studi di Perugia, ITALY

## Abstract

During the last years, many research efforts have been devoted to the definition of Fall Detection Systems (FDSs) that benefit from the inherent computing, communication and sensing capabilities of smartphones. However, employing a smartphone as the unique sensor in a FDS application entails several disadvantages as long as an accurate characterization of the patient’s mobility may force to transport this personal device on an unnatural position. This paper presents a smartphone-based architecture for the automatic detection of falls. The system incorporates a set of small sensing motes that can communicate with the smartphone to help in the fall detection decision. The deployed architecture is systematically evaluated in a testbed with experimental users in order to determine the number and positions of the sensors that optimize the effectiveness of the FDS, as well as to assess the most convenient role of the smartphone in the architecture.

## 1. Introduction

The definition of reliable, automatic and cost effective Fall Detection Systems (FDSs), which can profit from the latest advances in the field of electronics, signal processing and wireless communications, has become a popular research topic during the last decade.

Falls are a significant cause of morbidity and mortality among the people over age 64 [1], as well as an important source of expenditure for the health system in Western countries [2], with medical costs that are projected to reach $67.7 billion by 2020 only in the USA [3]. An immediate response after a collapse has been shown to be key to soften the most serious consequences of falls [4], including the FoF (Fear of Falling) syndrome. Additionally, FDSs are not only interesting for the elderly but also for different sectors of the population with a high risk of experiencing falls during professional or leisure activities in their daily life (e.g. aerial technicians, firefighters, mountain climbers, cyclists, etc.).

Wearable FDSs can be considered a sub-type of Body Area Networks (BANs). When compared with other wireless networks of battery-driven nodes, BANs pose specific problems. While scalability and Quality of Service (QoS) may be of major importance for wide area sensor networks (such as those deployed for smart cities [5]) and Vehicular Cloud Services [6], energy-limited wireless backbones [7] or data-centers for Big Data Stream Mobile Computing [8], human factors and ergonomics are essential issues for an adequate design of a BAN.

In contrast with other types of BAN, which incorporate particular biosignal sensors, such as thermometers, electrocardiography, SpO2 or plethysmography [9] sensors, which are normally connected to a holter monitor, wearable FDSs employ transportable mobility sensors (normally accelerometers but also gyroscopes and, more rarely, magnetometers). In addition a FDS requires computing power and memory resources to implement a fall detection algorithm as well as long-range communication interfaces to send the fall alarms (e.g. text messages, automatic voice calls, etc.) to a remote monitoring point. As all these features are already present in current smartphones at a relatively low cost, many recent works (see [10] or [11] for a full review on this subject) have been devoted to implement FDSs as simple mobile *apps* to be installed in these popular devices.

Most of these proposals are conceived under the idea of a ‘stand-alone’ product where the smartphone constitutes the only element in the system, simultaneously acting as sensor, wireless gateway and alerting unit as well as the hardware that processes the sensor measurements and generates the detection decision.

The main limitation of many of those studies is that the architecture and the detection technique are devised, optimized and tested assuming that the phone is worn (and even affixed with an elastic band) on a very particular body location. In [12] the best position for the smartphone in a ‘standalone’ smartphone-based FDS is investigated. Authors evaluates the developed FDS locating the phone on three different positions (thigh, chest and waist) concluding that the poorest accuracy is accomplished when the device is maintained in a thigh pocket. These unnatural placements (e.g. chest) of the phone obviously hinder a realistic application of smartphone-based FDSs, by not only altering the way in which the device is transported but also hampering the use of the conventional functions of the phone. Conversely, a natural but loose fitting attachment of the phone to the body may affect the efficacy of the detection process. In fact, authors in [13] show that the effectiveness of a FDS can noticeably diminish if the smartphone shakes inside the pocket.

A better trade-off between the efficacy of the FDS and ergonomics can be achieved if the system incorporates the information from other external wearable sensors. Nowadays, tiny, light and inexpensive sensing ‘motes’ integrating accelerometers and gyroscopes can be seamlessly transported (e.g. smartwatches) or easily integrated in the clothes as garment accessories. Many of these wearable motes natively integrate wireless communications standards, such as Bluetooth, which is also supported by commercial smartphones, so that they can cooperate to generate the classification decision required by the FDS.

Apart from those cases of multisensor data fusion (reviewed in [14]) where the data obtained through wearable devices are combined with the signals measured by the sensors (cameras, infrared sensors, microphones, etc.) in an external context-aware system, the use of BANs to characterize the human mobility has been extensively studied by the literature. In this field, there are several works [15] [16] [17] [18] [19] [20] [21] [22] that have evaluated the capability of a set of 3 to 6 accelerometers distributed through strategic points of the human body (normally chest, arm, wrist, thigh, hip, waist and/or ankle) to deploy automatic recognition systems of daily living activities. However, in these papers the possibility of considering falls as another identifiable activity is not considered. The system presented in [23] detects falls as abnormal human activities through a FDS that attaches three motes to the shoulder, waist and knee of the user, respectively. A similar system employing two Shimmer sensor platforms located at the chest and thigh is described in [24]. The FDS in [25] incorporates six on-body Xsens MTw sensors which are fixed with straps to the head, chest, waist, right wrist, right thigh, and right ankle of the user. The sensor network, which communicates wirelessly via ZigBee with a station connected to a PC, is employed to evaluate the performance of different detection techniques. Nonetheless the importance of the position of the sensors in the detection process is not investigated in these papers.

In [26] authors employ three accelerometers in the arms and chest of a set of volunteers to compare their estimation of the corporal physical stillness during meditation.

There has been much less research efforts in smartphone-based FDSs that incorporate on-body sensors apart from those embedded on the phone. A combination of a commercial smartwatch and a smartphone has been proposed in [27] [28][29] [30] to implement a FDS. In some cases the fall detection is the result of the combined decision of the measurements of both devices, but just the study in [29] assesses the added value provided by the inclusion of the smartwatch with respect to the ‘smartphone-only’ based solution. Similarly, authors in [31] describes a prototype of a FDS consisting in a smartphone (uncomfortably fastened to the chest) and an external Bluetooth-enabled accelerometer (attached to a thigh), although no experiments are executed to validate the system accuracy.

In this paper we describe a new prototype of FDS that combines the measurements from a smartphone and those captured by a group of wearable sensing motes. The goal of the prototype is to systematically assess the benefits of considering and combining the measurements obtained at different parts of the body.

The paper is organized as it follows: after presenting the state of the art in this introduction, section two explains the general architecture of the proposed system and the detection algorithms that will be used during the experimental phase. Section 3 describes the utilized testbed, summarizing and discussing the most relevant obtained results. Finally, the main conclusions are drawn and enumerated in Section 4.

## 2. Methods

**Fig 1:**
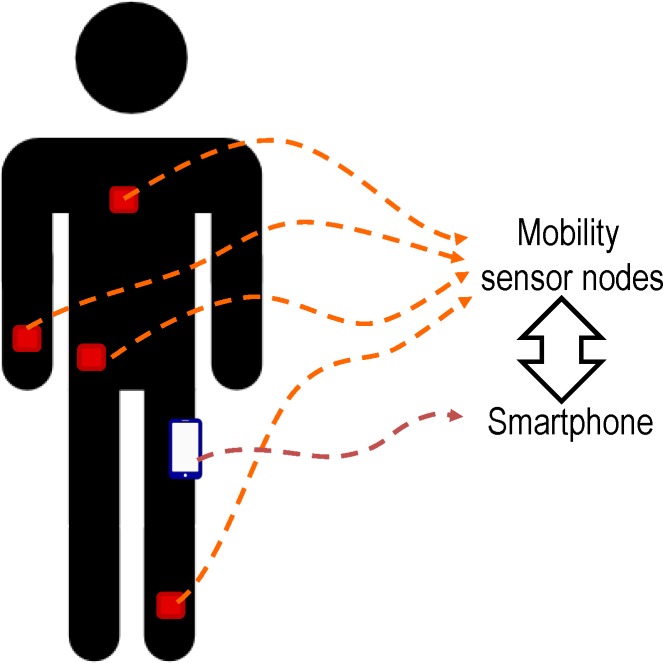
Basic Architecture of the Fall Detection System.

The deployed system is sketched in Fig 1. The system consists of an Android smartphone and a set of mobility sensors attached to different parts of the body. The sensors were implemented in SimpleLink Multi-Standard CC2650 SensorTag units of Texas Instruments. These motes (devised for a seamless and rapid design of IoT or Internet-of-Things applications) incorporate 10 different Microelectromechanical (MEMS) sensors, including a multi-chip MPU-9250 module by InvenSense, housing a tri-axis accelerometer, a triaxial gyroscope and a magnetometer. The motes, which are powered by a single CR2032 button cell battery, are based on a CC2650 ARM microcontroller with multi-standard support of low power wireless communications (Bluetooth Low Energy, ZigBee and 6LowPAN).

Taking advantage from the fact that most current smartphones natively include Bluetooth and Bluetooth Low Energy (BLE) interfaces, we designed a BLE star topology in which the smartphone performs as the central communication node of the architecture. Accordingly, the original software of the motes was reprogrammed to periodically activate the triaxial inertial sensors to sense the corresponding mobility magnitudes and then send them to the smartphone via BLE. An Android application (app) was implemented in the smartphone to receive and store the mobility samples (the 9 values measured by the tri-axial sensors) sent from the motes. In order to identify the source and timing of the measurements, for every received sample, the smartphone associates a timestamp and the Bluetooth MAC address of the mote that transmitted it. In addition, the app is also in charge of saving the signals that are periodically captured by the accelerometer, gyroscope and magnetometer of the smartphone. As a consequence, whenever the app is initiated, a single trace file containing the different samples that describe the movement is progressively created. This file is closed and the capture of the samples is interrupted after a pre-set time interval or as soon as the user presses a stop-button in the app.

This architecture is specifically conceived to obtain a realistic repository of multisensory mobility samples aimed at evaluating Fall Detection Algorithms. Following the methodology massively employed by the related literature, the repository is generated with the help of a group of experimental subjects, who systematically execute a predefined set of different ADLs (or Activities of Daily Living) and mimicked falls on a mattress. The validity of this procedure (healthy young adults emulating falls by tumbling on protective pads) to analyze the accuracy of FDSs mainly intended for older people has been largely discussed in works such as [32][33][34][35] or [36]. However, due to the inherent difficulties of acquiring a representative number of mobility traces of actual falls, this discussion is out of the scope of this paper.

Before any experiment begins, the user must enter into the app the corresponding information regarding the conditions of the test to be performed: identification and characteristics (age, height, weight and gender) of the experimental subject, nature of the movement to be emulated (ADL or fall and typology of fall or ADL), the position and the number of the trial (as long as the subjects execute the same movements several times). In turn, the app automatically obtains the features of the sensors that will capture the signals (vendor, range and resolution) as well as the employed sampling rates. All this information is saved at the beginning of the recording file where the mobility measurements will be recorded. In a second phase, these trace files resulting from the experiments, which are intended for the offline evaluation of fall detection algorithms, are retrieved from the smartphone memory and stored in a computer to further proceed with their analysis.

### 2.1. Fall Detection Algorithms

The deployed architecture is utilized to compare the performance of four different fall detection algorithms that make the detection decision as a function of the signals measured by the built-in accelerometers of the wearable devices. We compared four basic ‘thresholding’ algorithms, which only identify a fall if one or several mobility variables surpass some decision thresholds (simultaneously or in consecutive observation intervals). We just considered these simple and untrained methods as, in a real scenario of real-time-detection, sensor motes may present relevant constrains in terms of computational and storage resources, so that they may pose significant restrictions to the implementation of more sophisticated detection techniques (such as those based on artificial intelligence, rule-based or machine learning techniques) that have been utilized by the research literature (see [11] or [37] for a comprehensive state-of-the-art on smartphone-based FDSs). The four compared algorithms, also considered in [38] and [29], are briefly described in the following paragraphs.

#### Basic Threshold Monitoring

Falls cause the presence of unexpected peaks of the body acceleration. So, according to a basic thresholding method, a fall is assumed whenever the module of the acceleration (or *SMV*, Signal Magnitude Vector) exceeds a certain upper threshold (*SMV*_*Th*_). The value of *SMV*_*i*_ (for the *i*-th measurement of the acceleration module) can be computed as:

$${SMV}_{i}=\sqrt{{\left|A_{x_{i}}\right|}^{2}+{\left|A_{y_{i}}\right|}^{2}+{\left|A_{z_{i}}\right|}^{2}}\;m/s^{2}$$ (1)

where *A*_*xi*_, *A*_*yi*_ and *A*_*zi*_ define the three components of the acceleration vector for that *i*-th sample in the direction of the *x*, *y*, and *z*-axes, respectively. These components are periodically measured by the tri-axial accelerometer embedded in the smartphone and the external sensors.

#### Fall Index

This method, presented by Yoshida in [39], permanently estimates a metric called Fall Index (*FI*). The index *FI*_*i*_ (for the *i*-th measurement interval) can be calculated from the last 20 samples of the three components of the acceleration by applying the filter:

$${FI}_{i}=\sqrt{\sum_{k=x,y,z}\sum_{i-19}^{i}{\left(A_{k_{i}}-A_{k_{i-1}}\right)}^{2}}$$ (2)

where the sub-index *k* designates the axis (*x*,*y*,*z*) of the corresponding measured acceleration component.

As in the previous method, whenever a new sample is captured, *FI*_*i*_ is compared with a binary decision threshold (*FI*_*Th*_) to determine the fall occurrence.

This method tries to avoid the false positives generated by the basic thresholding technique when the body carries out some kind of brusque movements. Conversely, the algorithm is expected to be less robust in the presence of ‘slow’ falls, which may remain undetected.

#### PerFallD

This method is a variant of the algorithm described in [38]. The method performs the detection by monitoring the SVM and the module of acceleration at the absolute vertical direction (*|A*_*vi*_*|*), which can be computed (for the *i*-th sample) as:

$$\left|A_{v_{i}}|=|A_{x_{i}}sin\Phi_{i}+A_{y_{i}}sin\theta_{i}-A_{z_{i}}cos\theta_{i}cos\Phi_{i}\right|$$ (3)

In the previous formula *Φ*_*i*_ and *θ*_*i*_ and denote the measured values of the roll and pitch angles (at the *i-th* sampling interval) of the accelerometer. For the sake of simplicity, to estimate these angles, we assume a static condition in which the body is just subjected to the gravity force (which does not really hold for the case of a falling person). Under these circumstances, *Φ*_*i*_ and *θ*_*i*_ are estimated as [40]:

$$\Phi_{i}=\mathrm{atan}\;\left(\frac{A_{xi}}{\sqrt{A_{yi}^{2}+A_{zi}^{2}}}\right)$$ (4)

$$\theta_{i}=\mathrm{atan}\;\left(\frac{-A_{yi}}{A_{zi}}\right)$$ (5)

The algorithm constantly examines if the absolute maximum difference of the captured values of *SMV*_*i*_ within a short sliding time window (of duration *win*_*P*_) surpasses a triggering threshold (*SMV*_*ThP*_), which could indicate that the user’s body has hit the floor. A similar criterion is applied in parallel with the values of |*A*_*vi*_| and the corresponding thresholds *AV*_*ThP*_. A fall is only detected if the two detection conditions are simultaneously satisfied for *SVM* and |*A*_*vi*_| for the same sliding time window.

#### iFall (Two-phase)

This method, which is a simplified version of iFall, which was presented in [41], assumes the existence of two consecutive stages or phases: free fall and impact. The first one is detected with the sharp decrease (or negative peak) of the acceleration module, which is associated to a free fall. After this phase, the impact against the floor is again assumed to produce a brusque peak of the acceleration. Consequently, the method identifies a fall occurrence if the value of the Signal Magnitude Vector consecutively goes beyond a lower (*SMV*_*l*_) and an upper threshold (*SMV*_*u*_) during a pre-set observation time window (of duration *win*_*O*_). For this purpose the evolution of the signal *SMV*_*i*_ is permanently tracked. If the module of the acceleration vector falls at any sample under the threshold *SMV*_*l*_, a free-fall phase is presumed. In that case, the method checks if the impact takes place by comparing *SVM*_*i*_ with the upper threshold *SMV*_*u*._ If this upper threshold is surpassed at any instant during a time window of duration *win*_*O*_ following the detection of the free fall, a fall is assumed to have occurred. In other case, after an observation window without detecting the impact, the fall occurrence is discarded, the algorithm returns to the initial point and the module *SMV*_*i*_ is again compared with the lower detecting threshold *SMV*_*l*_ in order to identify a new fall phase.

### Ethics Statements

The individual in this manuscript has given written informed consent (as outlined in PLOS consent form) to publish these case details

## 3. Results and Discussion

The proposed architecture was devised to evaluate the importance of the number and position of the wearable mobility sensors when characterizing the human mobility to discriminate falls from ADLs. For that purpose, a set of systematical experiments were executed to capture the mobility traces of a wide set of experimental users while emulating falls and ADLs.

17 healthy experimental subjects participated in the tests to acquire a significant number of mobility samples: 6 females and 11 males, aged from 14 to 55, with weights between 50 and 93 kg and heights ranging from 155 to 195 cm.

During the tests the subjects transported the smartphone in a trouser pocket (which can be considered a ‘natural’ position for this device) while four TI SensorTags were attached (with elastic bands) to four different locations in the body: the left wrist, the waist, the chest and the ankle. This positions (specially pocket-or thigh-, waist and chest) include the most typical sensor placements that are commonly considered by the related literature (see [37] or [42] for a further analysis of this matter).

Two different smartphones were employed in the testbed: a Samsung S5 (which embeds an Invesense MPU6500 motion tracking chip with a range of ±2 g for the acceleration sensor) and a LG G4 device (which incorporates a Bosch LGE Accelerometer with a ±16 g range). The sampling rate in the smartphone was set to the maximum (200 Hz). Due to the limitations in the mote hardware and in the Bluetooth communications, the employed sampling rate in the SensorTags was 20 Hz. By using this data rate, during the tests, no congestion in the star topology formed by the smartphone and the four external accelerometers was observed. So, all the packets generated by the sensors were correctly received and stored by the app in the smartphone with negligible losses.

**Fig 2:**
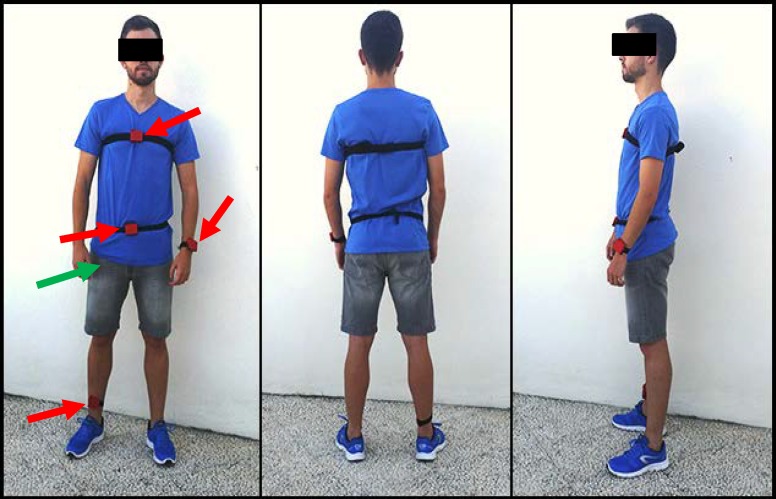
Experimental subject with the multisensory system.

A snapshot of one of the experimental subjects wearing the multisensor system is included in Fig 2 (the motes are highlighted with a red arrow while the position of the smartphone is marked with a green arrow). All the tests were carried out in a domestic environment (a bedroom or a terrace).

Every subject executed a set of 7 predetermined ordinary ADLs: normal walking, light jogging, body bending, hopping, climbing stairs (up and down), lying down (and getting up) on (from) a bed, and sitting down (and up) on (from) a chair. In addition all the subjects except two (those who were older than 50 years) emulated 3 different categories of falls (lateral, frontal and backwards) on a mattress. For both ADLs and simulated falls, the initial position of the subjects was standing straight up. Every participant repeated each movement at least 3 times. As a result of the experiments, 530 valid samples, in the format of CSV (comma-separated-values) files, containing the periodical measurements of the 5 sensors (the IMU of the Smartphone and the 4 SensorTag motes) were obtained and stored in the internal memory of the smartphone. All the mobility traces that were utilized for this study (as well as some video-clips describing the experimental testbed and the executed ADLs and falls) are fully and publicly available in our web sites (see [43] or [44] for further details).

The data files were used for the offline assessment of the performance of the aforementioned fall detection algorithms, which were implemented through simple Matlab scripts [45]. Therefore, the algorithms were evaluated with the same mobility patterns. As the effectiveness of the algorithms to distinguish falls from ADLs clearly relies on the election of the different decision thresholds, we checked the performance of the algorithms for a wide range of values of the decision thresholds. In particular, we modified the value of the most critical decision threshold, the upper value of the acceleration module: *SMV*_*Th*_ (for basic thresholding), *FI*_*Th*_ (for *Fall Index* algorithm), *SMV*_*ThP*_ and *AV*_*ThP*_ (for the two-phase detection), and *SMV*_*u*_ (for *iFall* algorithm). The rest of the parameters of the two last algorithms were established based upon different initial tests to maximize the performance metrics: *win*_*P*_ = 0.5 s (for PerFallD detection), *win*_*O*_ = 0.5 s and *SMV*_*l*_ = 0.65 g (6.37 m/s^2^) (for *iFall* algorithm). In any case, these secondary parameters have a relatively much lower impact on the results.

As the quality metrics we employed three parameters that are generally considered by the research on FDSs (and in the generic evaluation of pattern recognition systems with binary decision):

- *Sensitivity* (*Se*), defined as the ratio between the number of true positive or TP (falls that were correctly identified) and the number of falls that actually occurred, i.e. the sum of true positives and false negatives (FN) (or falls wrongly classified as ADLs).
- *Specificity (Sp)*, which can be similarly computed as the ratio between the measured amount of TN or true negatives (properly identified ADLs) and the total number of executed ADLs, i.e. TN plus FP (False Positives or ADLs misidentified as falls).

In any system intended for binary pattern classification, a trade-off between these two parameters (*Sp* and *Se*) must be achieved as long as they strongly depend on the decision thresholds in an opposite way (e.g. a more restrictive threshold normally increases *Sp* while it reduces *Se*). Thus, after testing the algorithms with the whole range of contemplated decision thresholds, we utilized as a related performance metric the maximum obtained value of the geometric mean of *Se* and *Sp*.

- The third considered metric is the AUC *(Area Under Curve*) or c-statistic. This parameter is calculated from the Receiver Operating Characteristic (ROC), or ROC curve, which is in turn obtained by plotting *Se* versus the False Positive Rate (*1-Sp*) for the different values of the discrimination threshold. The AUC (which is linked to the probability of a having an adequate classification of a sample) is numerically estimated by trapezoidal approximations.

In addition, we considered two new particular metrics derived from the analysis of *Se* and *Sp*. In most papers in the literature, the importance of *Se* and *Sp* (that is to say, the capacity to correctly recognize falls and ADLs) is assumed to be the same. However, the designer of a FDS should take into account that falls are rare events while ADLs are continuously taking place. A value of 0.90 for *Se* could be considered acceptable as far as, on average, only one out of ten falls would be misidentified. On the contrary, a *Sp* of 0.9 implies that one out of ten ADLs will generate a false positive and, consequently, a false alarm. These false alarms would probably annoy the monitored user (if he/she has the chance to deactivate the emission of the alert to the remote monitoring point) or would cause unnecessary concern amongst the caregivers. As dozens (or hundreds) of ADLs are expected to be executed every day by the target population of these systems, a *Sp* lower than 0.9 will entail the occurrence of several false alarms every day. As a consequence, in the analysis of the algorithms, we calculate the maximum sensitivity that is achieved when a minimum mandatory specificity is established. In particular, we offer the results of the maximum obtained *Se* when the selected thresholds yield a minimum *Se* of 0.9 and 0.95.

Table 1 includes the results of these four quality metrics (AUC, maximum achieved geometric mean of specificity and sensitivity, and maximum achieved sensitivity for a minimum specificity higher than 0.9 and 0.95) when the four aforementioned thresholding methods are applied to the captured samples of ADL and emulated falls.

**Table 1:** Comparison of the obtained results for the different detection algorithms (considering all the samples) as a function of the sensors considered to generate the detection decision: BT (Basic Thresholding), iFall, FI (Fall Index), PerF (PerFallD).

*Smartphone*	Considered Sensor Motes (SensorTags)				AUC				$max(\sqrt{Se\cdot Sp})$				*Se*_*max*_(*Sp*>0.90)				*Se*_*max*_(*Sp*>0.95)			
Thigh	Chest	Waist	Wrist	Ankle	BT	FI	PerF	iFall	BT	FI	PerF	iFall	BT	FI	PerF	iFall	BT	FI	PerF	iFall
✓					0.854	0.738	0.844	0.727	0.801	0.705	0.788	0.804	0.349	0.211	0.354	0.349	0.187	0.134	0.153	0.187
	✓				0.831	0.835	0.809	0.651	0.802	0.832	0.795	0.798	0.220	0.239	0.191	0.220	0.139	0.120	0.129	0.139
		✓			0.877	0.811	0.849	0.582	0.847	0.764	0.835	0.784	0.469	0.316	0.278	0.383	0.234	0.144	0.167	0.196
			**✓**		**0.935**	**0.948**	**0.914**	**0.710**	**0.858**	**0.886**	**0.861**	**0.765**	**0.713**	**0.756**	**0.608**	**0.574**	**0.545**	**0.608**	**0.411**	**0.440**
				✓	0.877	0.812	0.850	0.580	0.847	0.764	0.835	0.784	0.469	0.316	0.278	0.383	0.234	0.144	0.167	0.196
✓	✓				0.845	0.790	0.815	0.622	0.812	0.761	0.810	0.802	0.368	0.187	0.273	0.359	0.148	0.139	0.148	0.148
✓		✓			0.854	0.738	0.844	0.726	0.801	0.705	0.788	0.804	0.349	0.211	0.354	0.349	0.187	0.134	0.153	0.187
**✓**			**✓**		**0.918**	**0.798**	**0.904**	**0.652**	**0.863**	**0.749**	**0.868**	**0.791**	**0.622**	**0.359**	**0.526**	**0.498**	**0.455**	**0.211**	**0.368**	**0.373**
✓				✓	0.854	0.738	0.844	0.727	0.801	0.705	0.788	0.804	0.349	0.211	0.354	0.349	0.187	0.134	0.153	0.187
✓	✓	✓			0.845	0.790	0.815	0.622	0.812	0.761	0.810	0.802	0.368	0.187	0.273	0.359	0.148	0.139	0.148	0.148
✓	✓		✓		0.877	0.812	0.850	0.580	0.847	0.764	0.835	0.784	0.469	0.316	0.278	0.383	0.234	0.144	0.167	0.196
✓	✓			✓	0.845	0.790	0.815	0.622	0.812	0.761	0.810	0.802	0.368	0.187	0.273	0.359	0.148	0.139	0.148	0.148
✓		✓	✓		0.918	0.798	0.904	0.652	0.863	0.749	0.868	0.791	0.622	0.359	0.526	0.498	0.455	0.211	0.368	0.373
✓		✓		✓	0.854	0.738	0.844	0.726	0.801	0.705	0.788	0.804	0.349	0.211	0.354	0.349	0.187	0.134	0.153	0.187
✓			✓	✓	0.918	0.798	0.904	0.652	0.863	0.749	0.868	0.791	0.622	0.359	0.526	0.498	0.455	0.211	0.368	0.373
✓	✓	✓	✓		0.877	0.812	0.850	0.580	0.847	0.764	0.835	0.784	0.469	0.316	0.278	0.383	0.234	0.144	0.167	0.196
✓	✓	✓		✓	0.845	0.790	0.815	0.622	0.812	0.761	0.810	0.802	0.368	0.187	0.273	0.359	0.148	0.139	0.148	0.148
✓	✓		✓	✓	0.877	0.812	0.850	0.580	0.847	0.764	0.835	0.784	0.469	0.316	0.278	0.383	0.234	0.144	0.167	0.196
✓		✓	✓	✓	0.918	0.798	0.904	0.652	0.863	0.749	0.868	0.791	0.622	0.359	0.526	0.498	0.455	0.211	0.368	0.373
	✓	✓			0.831	0.835	0.809	0.651	0.802	0.832	0.795	0.798	0.220	0.239	0.191	0.220	0.139	0.120	0.129	0.139
	✓		✓		0.867	0.872	0.847	0.595	0.843	0.867	0.834	0.784	0.426	0.445	0.287	0.335	0.172	0.167	0.144	0.139
	✓			✓	0.831	0.835	0.809	0.651	0.802	0.832	0.795	0.798	0.220	0.239	0.191	0.220	0.139	0.120	0.129	0.139
		**✓**	**✓**		**0.935**	**0.948**	**0.914**	**0.711**	**0.858**	**0.886**	**0.861**	**0.765**	**0.713**	**0.756**	**0.608**	**0.574**	**0.545**	**0.608**	**0.411**	**0.440**
		✓		✓	0.877	0.811	0.849	0.582	0.847	0.764	0.835	0.784	0.469	0.316	0.278	0.383	0.234	0.144	0.167	0.196
			**✓**	**✓**	**0.935**	**0.948**	**0.914**	**0.711**	**0.858**	**0.886**	**0.861**	**0.765**	**0.713**	**0.756**	**0.608**	**0.574**	**0.545**	**0.608**	**0.411**	**0.440**
	✓	✓	✓		0.867	0.872	0.847	0.595	0.843	0.867	0.834	0.784	0.426	0.445	0.287	0.335	0.172	0.167	0.144	0.139
	✓	✓		✓	0.831	0.835	0.809	0.652	0.802	0.832	0.795	0.798	0.220	0.239	0.191	0.220	0.139	0.120	0.129	0.139
	✓		✓	✓	0.867	0.872	0.847	0.595	0.843	0.867	0.834	0.784	0.426	0.445	0.287	0.335	0.172	0.167	0.144	0.139
		✓	✓	✓	0.935	0.948	0.914	0.711	0.858	0.886	0.861	0.765	0.713	0.756	0.608	0.574	0.545	0.608	0.411	0.440
	✓	✓	✓	✓	0.867	0.872	0.847	0.595	0.843	0.867	0.834	0.784	0.426	0.445	0.287	0.335	0.172	0.167	0.144	0.139
✓	✓	✓	✓	✓	0.877	0.812	0.850	0.580	0.847	0.764	0.835	0.784	0.469	0.316	0.278	0.383	0.234	0.144	0.167	0.196

In order to analyze the importance of the position of each body sensor, the algorithms were selectively applied depending on the sensors that were considered to determine the fall decision. As the system includes five mobility sensors (the smartphone in the thigh pocket and the four SensorTag motes attached to the user’s chest, waist, wrist, and ankle) up to 31 (2^5^−1) different possible combinations were investigated. For every analyzed combination and every tested experiment, a fall is identified only if all the considered sensors individually classify the activity as a fall occurrence, that is to say, if the application of the corresponding algorithm to the signals captured by all the contemplated sensors produces a positive. Other combination policies (e.g. the decision of a positive if just one sensor detects a fall) were disregarded with the goal of minimizing the false positive rate and hence maximizing the specificity. For each studied combination, Table 1 indicates the considered sensors with a ‘tick’ sign (✓). Thus, the first five rows in the table would correspond to the hypothetical situation where just one single sensor is worn by the monitored user. Conversely the last row in the table refers to the case where the individual decisions of all the five mobility sensors are taken into consideration to generate the global classification of the system.

The table clearly shows that, for all metrics, the best results are obtained for those combinations (highlighted in bold characters) where the decision is based on the signals captured by the device attached to the wrist. The abrupt involuntary actions of the hands to protect the body from the impact of the fall could explain this behavior as they produce a sudden anomalous mobility pattern of the arms. The main problem of using the wrist as the unique reference point of the FDS is that hands enjoy a great freedom of movement with respect to the rest of the body. In an actual scenario of application of the FDS, in order to prevent false positive induced by any arbitrary and brusque action of the arms (not related to falls), the use of sensors which are closer to the center of the body mass is advisable. In this sense, Table 1 also reveals that the combination of the decision of the wrist sensor and that of the device on the waist or the chest does not deteriorate the system performance. Conversely, the utilization of the sensors at the ankle and the smartphone does not bring any improvement in the detection process. Similarly the combination of the decisions produced by more than two sensors also degrades the performance metrics. These results are coherent with the conclusions of the study in [17], where it is shown that using more than two sensors in a classifier of human movements had no significant impact on the system efficiency. Authors in [18] also conclude that two IMU sensors can be sufficient to distinguish simple human daily activities.

As it refers to the compared algorithms, paradoxically, the basic thresholding and Fall Index methods yield the best outcome for practically all the tested combinations. Thus, in the light of the results, the increase of the computing complexity and the higher number of parameters required by PerFallD and, more specifically, by iFall (which considers two-phase in the analysis of the movements) are not justified.

In any case, Table 1 illustrates the inability of these threshold-based methods to achieve a reasonable sensitivity when a minimum specificity is required. For the best performing combination (wrist and waist or wrist and chest) and algorithm (Fall Index), a poor sensitivity just slightly higher than 0.6 (60%) is obtained for a specificity of at least 0.95 (i.e. when at least 95% of the ADLs are correctly classified).

**Fig 3:**
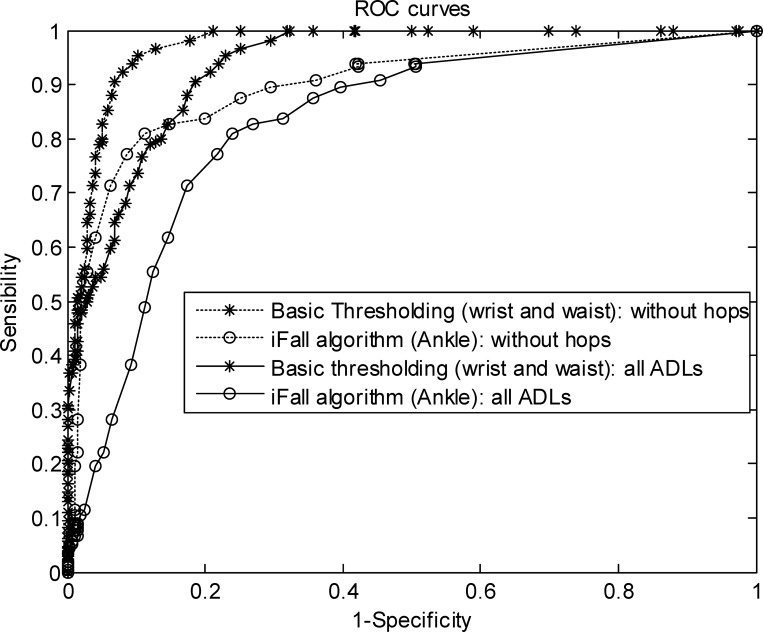
ROC curves obtained for the best and worst combinations of sensor positions and algorithms for the cases where samples generated by hops are included and excluded from the test ADLs.

However, a closer analysis of the experiments allows noticing that the measured performance of any FDS is strongly linked to the particular typology of the ADLs that are utilized to assess the accuracy of the system. In the case of our testbed, results are completely determined by the ineffectiveness of the methods to distinguish hops (one of the tested ADLs) from falls. Even in the limit case in which the decisions of the sensors in the five positions are considered (which is the most restrictive combination to produce false alarms), between 46% and 55% of the ADLs consisting of hops are misidentified as falls. As hopping is not a common activity among an important fraction of the target population of FDSs (older people), we repeated the previous performance analysis but now excluding the samples generated by hopping from the group of the ADLs. Table 2 sets out the system metrics obtained under these circumstances. Likewise, Fig 3 depicts the measured ROC curves for the best and worst sensor positions and algorithms both when samples generated by hopping are considered or excluded from the ADL set. The new results clearly evidence the improvement achieved for all the methods and sensor combinations when the evaluation tests do not include hops.

**Table 2:** Comparison of the obtained results for the different detection algorithms (without considering the ADL samples generated by hopping) as a function of the considered sensors: BT (Basic Thresholding), iFall, FI (Fall Index), PerF (PerFallD).

*Smartphone*	Considered Sensor Motes (SensorTags)				AUC				$max(\sqrt{Se\cdot Sp})$				*Se*_*max*_(*Sp*>0.90)				*Se*_*max*_(*Sp*>0.95)			
Thigh	Chest	Waist	Wrist	Ankle	BT	FI	PerF	iFall	BT	FI	PerF	iFall	BT	FI	PerF	iFall	BT	FI	PerF	iFall
✓					0,908	0,787	0,904	0,844	0,851	0,787	0,851	0,788	0,560	0,254	0,598	0,354	0,344	0,187	0,311	0,153
	✓				0,904	0,919	0,894	0,809	0,868	0,919	0,861	0,795	0,675	0,684	0,545	0,191	0,464	0,407	0,344	0,129
		✓			0,942	0,875	0,938	0,849	0,915	0,875	0,902	0,835	0,871	0,517	0,837	0,278	0,789	0,316	0,608	0,167
			**✓**		**0,971**	**0,976**	**0,968**	**0,914**	**0,925**	**0,976**	**0,930**	**0,861**	**0,938**	**0,967**	**0,947**	**0,608**	**0,789**	**0,861**	**0,770**	**0,411**
				✓	0,942	0,876	0,939	0,850	0,915	0,876	0,902	0,835	0,871	0,517	0,837	0,278	0,789	0,316	0,608	0,167
✓	✓				0,908	0,854	0,899	0,815	0,878	0,854	0,877	0,810	0,694	0,459	0,574	0,273	0,368	0,144	0,273	0,148
✓		✓			0,908	0,787	0,904	0,844	0,851	0,787	0,851	0,788	0,560	0,254	0,598	0,354	0,344	0,187	0,311	0,153
**✓**			**✓**		0,968	0,855	0,965	0,904	0,930	0,855	0,937	0,868	0,952	0,483	0,967	0,526	0,708	0,359	0,737	0,368
✓				✓	0,908	0,787	0,904	0,844	0,851	0,787	0,851	0,788	0,560	0,254	0,598	0,354	0,344	0,187	0,311	0,153
✓	✓	✓			0,908	0,854	0,899	0,815	0,878	0,854	0,877	0,810	0,694	0,459	0,574	0,273	0,368	0,144	0,273	0,148
✓	✓		✓		0,942	0,877	0,939	0,850	0,915	0,877	0,902	0,835	0,871	0,517	0,837	0,278	0,789	0,316	0,608	0,167
✓	✓			✓	0,908	0,854	0,899	0,815	0,878	0,854	0,877	0,810	0,694	0,459	0,574	0,273	0,368	0,144	0,273	0,148
✓		✓	✓		0,968	0,855	0,965	0,904	0,930	0,855	0,937	0,868	0,952	0,483	0,967	0,526	0,708	0,359	0,737	0,368
✓		✓		✓	0,908	0,787	0,904	0,844	0,851	0,787	0,851	0,788	0,560	0,254	0,598	0,354	0,344	0,187	0,311	0,153
✓			✓	✓	0,968	0,855	0,965	0,904	0,930	0,855	0,937	0,868	0,952	0,483	0,967	0,526	0,708	0,359	0,737	0,368
✓	✓	✓	✓		0,942	0,877	0,939	0,850	0,915	0,877	0,902	0,835	0,871	0,517	0,837	0,278	0,789	0,316	0,608	0,167
✓	✓	✓		✓	0,908	0,854	0,899	0,815	0,878	0,854	0,877	0,810	0,694	0,459	0,574	0,273	0,368	0,144	0,273	0,148
✓	✓		✓	✓	0,942	0,877	0,939	0,850	0,915	0,877	0,902	0,835	0,871	0,517	0,837	0,278	0,789	0,316	0,608	0,167
✓		✓	✓	✓	0,968	0,855	0,965	0,904	0,930	0,855	0,937	0,868	0,952	0,483	0,967	0,526	0,708	0,359	0,737	0,368
	✓	✓			0,904	0,919	0,894	0,809	0,868	0,919	0,861	0,795	0,675	0,684	0,545	0,191	0,464	0,407	0,344	0,129
	✓		✓		0,942	0,954	0,940	0,847	0,911	0,954	0,900	0,834	0,871	0,957	0,837	0,287	0,809	0,823	0,636	0,144
	✓			✓	0,904	0,919	0,894	0,809	0,868	0,919	0,861	0,795	0,675	0,684	0,545	0,191	0,464	0,407	0,344	0,129
		**✓**	**✓**		**0,971**	**0,976**	**0,968**	**0,914**	**0,925**	**0,976**	**0,930**	**0,861**	**0,938**	**0,967**	**0,947**	**0,608**	**0,789**	**0,861**	**0,770**	**0,411**
		✓		✓	0,942	0,875	0,938	0,849	0,915	0,875	0,902	0,835	0,871	0,517	0,837	0,278	0,789	0,316	0,608	0,167
			✓	✓	0,971	0,976	0,968	0,914	0,925	0,976	0,930	0,861	0,938	0,967	0,947	0,608	0,789	0,861	0,770	0,411
	✓	✓	✓		0,942	0,954	0,940	0,847	0,911	0,954	0,900	0,834	0,871	0,957	0,837	0,287	0,809	0,823	0,636	0,144
	✓	✓		✓	0,904	0,919	0,894	0,809	0,868	0,919	0,861	0,795	0,675	0,684	0,545	0,191	0,464	0,407	0,344	0,129
	✓		✓	✓	0,942	0,954	0,940	0,847	0,911	0,954	0,900	0,834	0,871	0,957	0,837	0,287	0,809	0,823	0,636	0,144
		✓	✓	✓	0,971	0,976	0,968	0,914	0,925	0,976	0,930	0,861	0,938	0,967	0,947	0,608	0,789	0,861	0,770	0,411
	✓	✓	✓	✓	0,942	0,954	0,940	0,847	0,911	0,954	0,900	0,834	0,871	0,957	0,837	0,287	0,809	0,823	0,636	0,144
✓	✓	✓	✓	✓	0,942	0,877	0,939	0,850	0,915	0,877	0,902	0,835	0,871	0,517	0,837	0,278	0,789	0,316	0,608	0,167

## 4. Conclusions

Last decade has witnessed a considerable number of research efforts that propose to utilize smartphones as fall detectors, benefiting from the low cost, the communication interfaces and the computing and sensing capabilities which are provided by these personal daily life devices. However, in order to attain an accurate characterization of the user mobility, the use of a smartphone as a wearable fall detection sensor obliges to transport it in an unnatural way. This paper has presented an experimental prototype of a smartphone-based FDS that incorporates several wireless sensor motes that can be attached to different part of the body. In contrast with most studies in the literature, which base their detection decision on the measurements of a single device, the proposed multisensor system permits to classify the movements as ADL or falls depending on a combined analysis of the signals captured by different accelerometers. The system has been utilized to investigate the accuracy of diverse detection algorithms as a function of the number and positions of the employed sensors. The testbed was deployed by the systematic execution of a set of ADLs and emulated falls by a wide group of experimental subjects. From the results and experience acquired with the tests, we can draw the following conclusions:

- The accuracy of the fall detection seems not to necessarily improve as the number of sensing devices increases. At least when a simple thresholding strategy and a basic combination policy are applied, using sensors in more than two positions of the human body provokes a degradation of the sensitivity, which is not compensated by a sufficient increase in the specificity.
- Most favorable results are clearly obtained when the mobility of the wrist is considered. To avoid ‘artifacts’ caused by sudden movements of the arms, the analysis indicates that the combination of a wrist sensor with a sensor in the chest or the waist could be a good option. The combination wrist/waist (or wrist/chest) clearly benefits from the simultaneous characterization of the actions exercised by the arms (especially intense during a fall occurrence to cushion the hit) and the mobility of the human trunk (where the body mass center is located). An important advantage of a FDS based on just two sensors at both the wrist and the waist is ergonomics, as it can be deployed more comfortably than other multisensory proposals. The accelerometer embedded in most present smartwatches or smartbands could be utilized as the wrist sensor whereas small wireless sensors integrating IMUs can be easily fixed to conventional belts without disturbing the user comfort.
- The evaluation testbed of a FDS must be carefully designed depending on the target public for whom the detection prototype is intended. ADLs generated as the results of sportive, brusque or physically demanding activities (emulated by young and healthy volunteers) can noticeably degrade the detection performance, but only under unrealistic circumstances of the use of the FDS. Thus, the design of the tested ADLs should be adapted to the typical daily activities of the target population of the system.
- The evaluation of a FDS must always take into account the high asymmetry in the occurrence of ADLs (dozens or hundreds per day) and falls (which are ‘rare’ events). As a consequence, the two typical metrics of these binary classification systems, that is to say, sensitivity and specificity, must not be considered at the same level when parameterizing the detection algorithms. The use of metrics such as AUC or the geometric mean of both statistics does not offer a meaningful insight into the actual performance of the system if we desire to evaluate it from a practical point of view. In our opinion, specificity (ability to discriminate a real ADL) must be paradoxically prioritized to avoid false alarms. Due to the high frequency of ADLs, common users will not accept FDSs unless a high specificity is guaranteed. Accordingly, in this paper, we have proposed and utilized a new metric to assess the performance of a FDS: the sensitivity (capacity to identify an actual fall) obtained when a minimum specificity is achieved. The parameters of the detection algorithms should be always tuned to yield this minimum specificity.
- The detection performed in the smartphone when it is transported in a ‘natural’ position (a trouser pocket in our testbed) does not increase the efficiency of the system when there are sensors on the wrist and waist. Thus, the detection function could be omitted at the smartphone, which could just be in charge of processing the signals (or alerts) from the wearable sensors and, in case of detecting a fall, sending the corresponding alarm to the remote monitoring point. This simplification of the role of the smartphone would avoid the extra battery drain demanded by the permanent monitoring of the embedded mobility sensors. Furthermore, the need of attaching this personal device to a particular point of the body would also be removed. The detection system would just require locating the smartphone in the vicinity of the wearable sensors to receive the acceleration measurements and/or the alarms. So, the user should be less concerned about the position of the smartphone as it could be transported in a purse or bag or even been located in an external and fixed point near the user (e.g. on a table) during home monitoring.
- Results show that Fall Index method and the simple use of a basic threshold outperform the effectiveness of other thresholding approaches. Anyhow, the performed massive tests illustrate the difficulties of basic threshold-based detection mechanisms to reach a sensitivity higher than 0.8 (80% of well identified falls) when a high specificity (95%) is requested. In future studies more complex algorithms (such as those presented in [46]) should be compared. Anyhow, whenever a new detection technique is proposed, authors should also evaluate if the limitations (in terms of battery consumption or computing power) of current smartphones or, in particular, sensor motes, pose any important restriction to the actual implementation of the algorithm on real devices. In addition, some sophisticated methods require supervised training to adapt the detection algorithm to the particular mobility patterns of the final user. The feasibility of this adaptation to individuals that cannot emulate or train certain physical activities (such as falls) should also be assessed. In any case, unlike most works in the literature on FDS, the numerous samples acquired during the evaluation of the prototype are available online at [43] and [44], so they can be used by any researcher for further analysis with new detection algorithms.

Besides, consumption and data congestion are key aspects for the viability of any wearable sensor network. In the deployed testbed, the employed data rate did not provoke any noticeable bottleneck in the smartphone. Thus, sensors were able to transmit the data through their corresponding BLE connections without detectable losses or significant delays. Similarly, the short duration of the tests did not pose any threat to the sensor’s batteries (which in turn benefit from the low consumption of BLE standard). However, future studies should thoroughly analyze the battery consumption in the motes and data congestion in the network under long-term monitoring or when higher sampling rates are considered. In this regard, the implementation of the detection algorithms in the motes could avoid the consumption caused by the periodical transmission of the acceleration coordinates. In that case, sensors should inform the smartphone only when a fall event is detected, which would drastically reduce the need for data exchange between the sensor and the smartphone. Conversely, if the algorithms are deployed in the sensor, the constrained storage and computing capabilities of the motes may hinder the use of complex detection techniques, which should be substituted by more basic algorithms as those employed in this paper.
